# High-Efficiency Screening of Monoclonal Antibodies for Membrane Protein Crystallography

**DOI:** 10.1371/journal.pone.0024653

**Published:** 2011-09-08

**Authors:** Hyun-Ho Lim, Yiling Fang, Carole Williams

**Affiliations:** Howard Hughes Medical Institute, Department of Biochemistry, Brandeis University, Waltham, Massachusetts, United States of America; University of South Florida College of Medicine, United States of America

## Abstract

Determination of crystal structures of membrane proteins is often limited by difficulties obtaining crystals diffracting to high resolution. Co-crystallization with Fab fragments of monoclonal antibodies has been reported to improve diffraction of membrane proteins crystals. However, it is not simple to generate useful monoclonal antibodies for membrane protein crystallography. In this report, we present an optimized process for efficient screening from immunization to final validation of monoclonal antibody for membrane protein crystallography.

## Introduction

Since the first high-resolution structure of a membrane protein, the photosynthetic reaction center, was solved in 1985 [1], more than 290 unique structures of membrane proteins have been reported (database for membrane proteins of known 3D structure, http://blanco.biomol.uci.edu/Membrane_Proteins_xtal.html). However, this represents only a tiny fraction of all membrane protein families. One of the biggest hurdles in membrane protein crystallography is to obtain crystals diffracting to high enough resolution for solving the structure. Crystallization of the membrane protein complexed with Fab or Fv fragments derived from monoclonal antibodies has, in a few cases, allowed new structures to be solved for ion channels, G-protein coupled receptors, and membrane transporters [2], [3], [4], [5], [6], [7], [8].

A crystallographically practicable antibody should meet three essential criteria. First, the hybridoma cells should secrete antibodies at a high level (50–100 mg/L culture). Second, the antibody must make a stable complex with the membrane protein in detergent solution. Finally, the protein-Fab complex should crystallize and diffract to ∼3 Å or better, to enable faithful model building. Screening of antibodies against discontinuous structural epitopes is typically performed using the experimental criteria of positive ELISA (Enzyme-linked immunosorbent assay) on membrane proteins in native conformations but negative western (or dot-blot) on SDS-denatured proteins [9], [10]. However, these criteria are not foolproof. Since the protein might retain partially folded structure in the western-blotting environment, so that discontinuous structural epitopes would score western-positive [11]. Although monoclonal Fab and Fv fragments have been used as powerful membrane protein crystallization chaperones, the procedures for obtaining useful monoclonal antibodies through conventional methods are time-consuming and costly, either in-house or by commercial production. Recent developments in phage and ribosomal display have resulted in faster screening procedures for antibody fragments and other crystallographic chaperones [12], [13]; however, they both involve proprietary reagents not yet commercially available.

**Figure 1:**
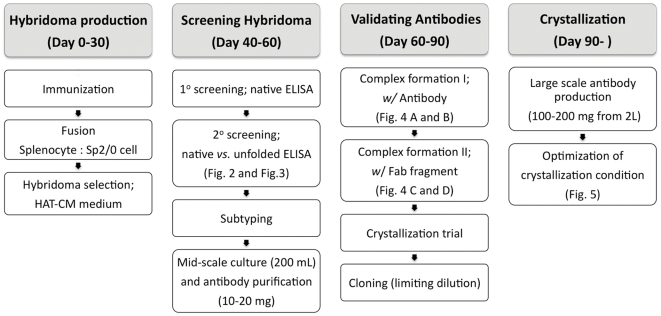
Experimental flowchart and estimated time line of monoclonal antibody screening for membrane protein crystallography.

Here, we describe a “backyard-factory” strategy for screening antibodies based on our experience in solving the structure of an Arginine-Agmatine exchange-transporter, AdiC [5], and crystallizing a bacterial CLC channel homologue, CLC-ec2. We have developed an efficient screening process requiring approximately three months from immunizing the mice to obtaining antibodies in sufficient quantity (10–20 mg) for initial crystallization trials (Fig. 1).

## Results and Discussion

### Immunization and initial screening

We tested five different preparations of membrane protein antigens for immunizing mice in order to compare immune responses to membrane proteins in different environments (in detergent micelles or reconstituted in liposomes) and different adjuvants [14], [15], [16], [17]: group 1, membrane protein in detergent micelles with Freund's adjuvant; group 2, proteoliposomes with lipid A (0.1 mg/ml) and aluminum hydroxide (1 mg/ml); group 3, proteoliposomes with plasmid DNA; group 4, proteoliposomes diluted in PBS; group 5, proteoliposomes with Freund's adjuvant. Although those immunization strategies have not been tested multiple times on different membrane proteins to make a solid conclusion, our results on two membrane proteins AdiC and CLC-ec2 indicate that mice immunized with group 1 and group 2 antigens gave a higher percentage of hybridoma cells secreting antibodies that bind to natively folded protein (Table 1). After evaluation of mouse sera by ELISA assay, splenocytes were isolated and fused with Sp2/0 cells to establish immortalized cell-lines. Ten to fourteen days after fusion, clumps of growing cells appeared in HAT-CM media, and tissue culture supernatants were screened to identify potentially high-secreting initial candidates.

**Table 1:** Statistics of screening antibodies.

		AdiC					CLC-ec2	
Immunization		group1	group2	group3	group4	group5	group1	group4
1° ELISA(Initial positives)		25	33	29	33	25	60	10
2° ELISA^1^(Native≫unfolded)		8/18	6/20	3/25	3/33	2/15	29/60	2/10
Subtypes	IgG1	0					6	
	IgG2a	10					7	
	IgG2b	7					12	
	IgG3	0					0	
	IgA	2					1	
	IgM	3					0	
	Not determined	0					5	
Complex formation^2^		22/22					12/31	

Legend: The Table reports results of hybridoma production for 7 separate immunizations – 5 with AdiC and 2 with CLC-ec2.

1 Displayed are the numbers of clones showing >2-fold brighter ELISA signals in native than unfolded conditions over the number tested.

2 Number of antibodies showing a chromatographically satisfactory complex over total tested.

**Figure 2:**
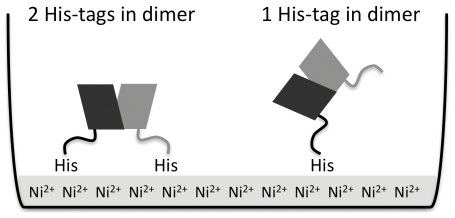
Schematic illustration of His-tagged membrane proteins on Ni^2+^-coated plate. Since AdiC and CLC-ec2 form stable dimers, a single His-tag system could be better to expose both *cis*- and *trans*-sides to the solution (*right*) than doubly tagged dimer (*left*).

Ni-NTA coated 96-well plates were used in ELISA assays for improved coating of our His-tagged proteins. Tandem dimer AdiC [5] and half His-tag removed CLC-ec2 were used for coating ELISA plate to expose as many epitopes as possible on both *cis* and *trans* side in these homodimeric membrane proteins (Fig. 2). In order to maintain a native state of membrane proteins throughout the ELISA assay, we included a detergent that was previously shown with functional reconstitution and chromatographic behavior to keep the protein properly folded. In the early stages of screening, it is important to identify hybridoma lines that secrete high levels of antibodies. Since crystal screens require a large quantity of the proteins, high yield is a prerequisite for crystallization trials.

### Screening antibodies that selectively bind to structural epitopes

**Figure 3:**
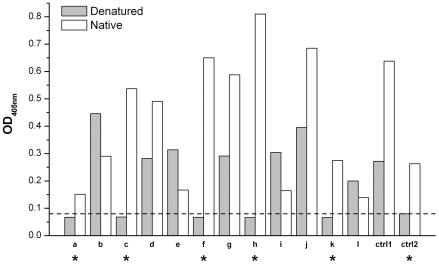
Representative ELISA results for the 12 initial candidates (a-l) for CLC-ec2 in the native or unfolded condition. Ctrl1 and Ctrl2 represent controls for test-bled serum (1/1000 dilution) on CLC-ec2 and anti-CLC-ec1 antibody (1 µg/mL concentration) on CLC-ec1, respectively. The dashed line indicates the cut-off line for negative result in the unfolded condition. Clones showing a negative signal in unfolded condition and that had a signal at least twice higher than background in the native conditions were to be tested further (marked as *).

Initial candidates with high signals (i.e. high secretion) were further tested in both native and unfolded conditions by ELISA to sort out antibodies specifically bound to the natively folded membrane proteins. To avoid partially folded structures in the western-blotting environment [11], protein antigens were denatured in 6 M guanidine-HCl and 0.1 M β-mercaptoethanol, before coating ELISA plates, and detergent was not used in this “unfolded ELISA” to prevent possible refolding. Previously validated antibodies against structural epitopes of the KcsA channel [3] and CLC-ec1 transporter [7] gave negative results in the “unfolded ELISA” and positive results in the “native ELISA”. These results strongly suggest that membrane proteins are unfolded under “unfolded ELISA” conditions and remain in the folded conformation under “native ELISA” conditions (Fig. 3).

We only chose clones showing near background signal in the “unfolded ELISA” and that had a signal in the native ELISA at least two-fold higher than background. For both of our test proteins, about 30% of antibodies were selected as specific against the native conformation (Table 1 and Fig. 2). These initial candidates were further scrutinized to select final cell lines. Although at this stage the cells are not yet cloned, our experience has shown that it saves significant time to assess antibody secretion and complex formation before establishing the hybridoma line.

### Purification strategy for different classes of immunoglobulin

Immortalized hybridoma cells secrete several distinct classes of immunoglobulin such as IgA, IgG and IgM molecules. In order to optimize the purification methods for each of these, we subtyped the initial candidates. Of 53 initial candidates, 3 were IgM, 3 were IgA and 42 were IgG (Table 1). Although it is possible that each uncloned initial candidate might contain more than one specific hybridoma, our results showed that such cases rarely occurred. After subtyping, we grew the initial candidates and purified antibodies from the supernatants using different chromatographic methods according to subtype. Initially, cells were grown for two weeks in 15-cm plates, each producing 200 mL of supernatant. All IgG subclasses (IgG1, IgG2a, IgG2b, and IgG3) were purified using protein G affinity columns. Since the Fc regions of IgA and IgM are hidden due to their multivalent structures, protein G columns cannot purify them. Instead, protein L affinity columns were used for IgA and IgM with kappa light chains, and hydroxyapatite for those with lambda light chains. Typically, a single plate yielded 5–20 mg of purified antibody.

### Evaluation of purified antibody

The ability of antibodies to form stable complexes was tested next. One major difficulty in membrane protein crystallization is due to the flexibility of its conformation, especially in the loops outside of the transmembrane region. These loops greatly affect the crystal packing. Fab acts as a chaperone stabilizing a certain conformation of the membrane protein, and also increases the polar surface of protein to facilitate packing [9]. Therefore, only tightly bound Fabs are suitable since weakly bound Fabs would increase heterogeneity of the crystallization mixture. For multi-transmembrane proteins, Fabs bound to several epitopes in different loops (discontinuous structural epitopes) can increase the structural stability of the protein tremendously leading high quality crystals.

**Figure 4:**
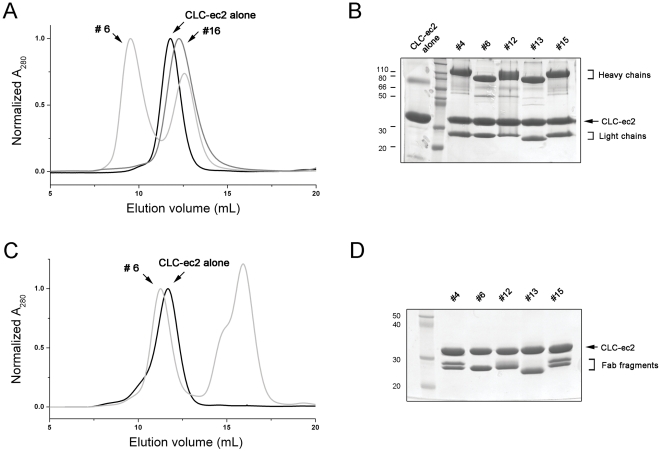
Evaluation of membrane protein-antibody complexes. A. Size-exclusion chromatography profiles of protein-antibody complexes. CLC-ec2 alone eluted at ∼12 mL (*black line*). Chromatogram of clone #6 showed two peaks at ∼9 mL for antibody-CLC-ec2 complex and at ∼13 mL for free antibody (*light gray line*). However, clone #16 eluted at ∼12.5 mL as a single broad peak (*gray line*), which represents co-migration of unbound antibody and CLC-ec2. B. Early peak fractions around 9–11 mL from size-exclusion column were run on SDS-PAGE. C. Fab#6-CLC-ec2 complex (*light gray line*) is eluted ∼0.5 mL earlier than CLC-ec2 alone (*black line*) followed by free-Fab and Fc in 15–16 mL position. D. Fab-CLC-ec2 complex fractions were resolved by SDS-PAGE.

A mixture of antibody and membrane protein in detergent micelles was run on a 24-mL Superdex 200 size-exclusion column. A useful protein-antibody complex elutes around 9–10 ml volume, 2–3 mL ahead of the membrane protein alone, with free antibody eluting at ∼12.5 ml (Fig. 4A and B). Stable antibody-protein complexes formed from all AdiC antibodies, but only less than half of CLC-ec2 antibodies (Table 1).

### Preparation of Fab-membrane protein complex and crystallization trial

In the final round of screening, Fab fragments are evaluated. We developed a quick procedure to test membrane protein-Fab complex formation without purifying the Fab fragment. First, it is necessary to assess the sensitivity of the membrane protein to papain cleavage. CLC-ec2 is not cleaved by papain, so antibodies for this protein were digested with soluble papain to produce a mixture of Fab/Fc fragments. However, AdiC is very susceptible to papain in trace amounts; we therefore used immobilized papain for producing Fab fragments in this case. After 2–6 hours of papain treatment, an excess of the Fab/Fc mix was combined with membrane protein and loaded onto a Superdex 200 column. The pure Fab/membrane protein complex elutes well ahead of both the membrane protein alone and the Fc fragment (Fig. 4C and D). The complex was concentrated to ∼10 mg/mL for crystallization trials. After initial crystallization trials, the promising candidates were established as stable monoclonals by limiting dilution. These lines were grown in 2 L gas permeable bottles to obtain >100-mg quantity of antibody for further optimization of diffraction quality.

**Figure 5:**
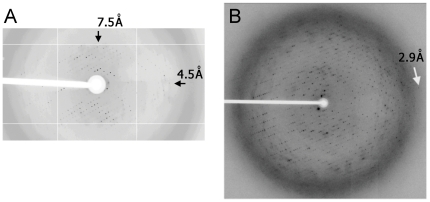
Improving diffraction patterns with one of the advantageous antibodies. A. Typical diffraction pattern of AdiC crystal. B. Diffraction pattern of AdiC-Fab21 complex.

Our experience in solving a AdiC-Fab complex structure has shown that among 22 antibodies that form complexes with AdiC and crystallize to diffracting crystals, 3 Fabs improved the resolution from anisotropic 4–7 Å to isotropic 3 Å resolution (Fig. 5). The structure was solved with Fab21-AdiC complex crystals [5].

### Concluding remarks

Structures of many membrane proteins have been solved without crystallographic chaperones; however, in cases of certain intractable membrane proteins, Fab fragment chaperones can lead to successful structure determination [4], [5], [6], [8]. Our experiences on a few membrane proteins have indicated that Fab is especially helpful for the proteins that can already form low resolution diffracting crystals. Comparing with many other techniques for membrane protein crystallization optimization such as “random” mutagenesis, homologue screen, detergent screen and mixture and so on, generating Fab is a “rational” approach, but used to be considered very time-consuming and costly. With our high-efficiency screening reported here, generating Fab for membrane protein crystallization has become quite manageable in a regular research lab.

## Materials and Methods

### Ethics statement

This study was performed in strict accordance with the Guide for the Care and Use of Laboratory Animals of the National Institutes of Health. The protocol was approved by the Institutional Animal Care and Use Committee of Brandeis University (Permit Number: 0809-43).

### Immunization and fusion

The standard immunization procedure was followed as described [17]. In short, 8-week-old BALB/c female mice were injected with 50 µg of antigen in 200 µl into the peritoneal cavity (intraperitoneal injections, *ip*). Two weeks after the first injection, mice were boosted with a second *ip* injection. A week later, mice were test-bled and the immunizations were evaluated using ELISA assay. Mice showing a robust immune response were further injected with both 2–5 µg of antigen into the tail vein (intravenous, *iv*) and 50 µg into the peritoneal cavity.

The mice were injected with the following five groups of different adjuvant conjugated antigens: group 1, membrane protein in detergent mixed with Freund's adjuvant (antigen∶adjuvant is 1∶1 (*v/v*)); group 2, proteoliposomes (membrane protein reconstituted into the lipid vesicles at the ratio of 100 µg/mg lipid in PBS buffer) mixed with lipid A (0.1 mg/ml) and aluminum hydroxide (1 mg/ml); group 3, proteoliposomes mixed with plasmid DNA (liposome∶DNA is 1∶1 (*v/v*), plasmid DNA concentration is 0.5 mg/ml); group 4, proteoliposomes diluted in PBS buffer without adjuvant (liposome∶PBS is 1∶1 (*v/v*)); group 5, proteoliposomes mixed with Freund's adjuvant (liposome∶adjuvant is 1∶1 (*v/v*)). Complete Freund adjuvant (CFA) for the first *ip* injection, and incomplete Freund adjuvant (IFA) for the following boost injections were used. In each group, two mice with the highest ELISA signal were given a third injection (both *ip* and *iv*). Note that for the groups with Freund as adjuvant, *iv* injections should not include Freud since it is very toxic and may kill the mice.

Two mice showing high ELISA signals from sera were sacrificed and spleens were isolated. Splenocytes were fused with the immortalized myeloma cell line Sp2/0-Ag14 (CRL-1581, ATCC) as previously described [17]. Briefly, splenocytes were mixed with Sp2/0 cell (3∶1 ratio), cells were spun, and 1 mL of 50% PEG1500 (*w/v*, Roche) was added to cell pellet drop-by-drop over 1 min. Then complete media (CM; 10% NCTC media (*v/v*), 2 mM glutamine, 1 mM sodium pyruvate, 1% MEM NEAA (*v/v*), 50 µg/mL gentamycin in DMEM (high glucose, Gibco)) was added to cells following sequences: 1 mL after 1 min, 2 mL after 2 min, 4 mL after 4 min and the final volume was brought to 15 mL. After spinning, the cells were resuspended in FBS and sat for 15 min. HAT-CM (CM with 10% hybridoma cloning factor (Roche) and 1× HAT supplement (Gibco)) was added to make final 20% FBS and cells were plated in 96 well plate at a density of 2×10^4^ Sp2/0 cells per well.

### ELISA and antibody subtyping

In order to evaluate hybridoma supernatants, ELISA assays were performed in native or unfolded conditions. His-tagged tandem dimer AdiC was expressed and purified as described [5]. CLC-ec2 was cloned in pASK90 vector with C-terminal His-tag, expressed in *E.coli* BL21 (DE3), and purified on Co-affinity column (BD Biosciences). The His-tag was completely removed by a 1-hr digestion with lysine endoproteinase C (LysC, 0.02 U/mg protein; Roche). For obtaining half His-tag removed CLC-ec2, proteolysis was performed for 0.5 hr with 1/10 of LysC (0.002 U/mg protein). Half His-tag removed CLC-ec2 was further purified on the size-exclusion chromatography and evaluated in SDS-PAGE. For ELISA under native conditions, His-tagged tandem dimer AdiC or half His-tag removed CLC-ec2 were diluted in PBS-DDM-BSA (PBS; 137 mM NaCl, 2.7 mM KCl, 10 mM Na_2_HPO_4_, 1.76 mM KH_2_PO_4_, pH 7.4, and 2 mM dodecyl-β-D-maltopyranoside (DDM, Anatrace) and 2 mg/mL BSA) at 20 µg/mL and 50 µL of protein (20 µg/mL) was incubated in each well of Ni-NTA HisSorb 96-well plates (Qiagen) for 1 hr at room temperature. After washing 3 times with PBS-DDM (1× PBS with 1 mM DDM), 50 µL of tissue culture supernatant and 50 µL of PBS-DDM-BSA were added to each well and incubated for 1 hr. Subsequently, wells were washed and incubated with a 1/2000 dilution of rabbit anti-mouse IgG antibodies (Promega) in PBS-DDM-BSA for 45 min. After washing 2 times with *p*-nitrophenyl phosphate (PNPP) reaction buffer followed by 3 washes with PBS-DDM, color development was initiated with 100 µL of PNPP (1 mg/mL). The reaction was stopped by addition of 50 µL of 2 M NaOH and the signal was read at 405 nm on a plate reader (Molecular Device). For unfolded conditions, membrane proteins (120 µg/mL) were treated with 6 M guanidine-HCl and 0.1 M β-mercaptoethanol at 90°C for 10 min, diluted six-fold with PBS and coated in Ni-NTA HisSorb plate. ELISA procedures were the same as native conditions except, without detergent. Antibody subtyping was done using Mouse Typer Sub-isotyping kit (Bio-Rad). Manufacturer's instructions were followed, except that using Ni-NTA coated plates were used and detergent was included in each step.

### Clonal selection

Monoclonal cell lines were established by limiting dilution [17]. Briefly, cells were diluted in CM-HAT with 20% FBS and 10% hybridoma cloning factor (Roche) at 0.25–1 cell per well and plated on 96-well plate. Supernatants were screened by ELISA after 10-day cultivation. Cells were subsequently transferred to 12-well, 6-well and 10-cm plates. After growing to confluence, FBS was lowered from 20% to 5% in 5% steps. For large scale production of monoclonal antibody, the monoclonal cells adapted in CM with 5% FBS were grown in two 15 cm-plate for 3–5 days, transferred in 2 L gas permeable culture bottles (Wilson Wolf Manufacturing). Two to four weeks after growing, antibodies were purified as described.

### Affinity purification of antibodies

Affinity purification on Protein G beads was used for IgG and on Protein L beads for IgM and IgA subclasses containing kappa light chains. Affinity resin (1 mL bed for 100 mL culture, Genscript) was packed in glass columns and the filtered tissue culture supernatant was loaded at 2–4 mL/min while optical density (OD) was monitored at 280 nm. The column was washed with PBS to baseline OD, and antibodies were eluted with 0.1 M glycine (pH 2.7). The eluate was immediately neutralized by addition of 0.2 volumes of 1 M tris-HCl, pH 8.0.

For IgM and IgA containing lambda light chain, hydroxyapatite columns were used. One gram of ceramic hydroxyapatite (Bio-Rad) was activated with 10 mL of 200 mM Na-phosphate, pH 8.0 and equilibrated in 10 mM Na_2_HPO_4_, pH 6.5. Before loading, Na_2_HPO_4_ was added to the tissue culture supernatant at final 5 mM and pH was adjusted to 6.5. After loading the sample and washing with equilibration buffer, proteins were eluted with two sequential linear gradients (40 column volumes each): equilibration buffer/high-salt buffer (2 M NaCl, 10 mM Na_2_HPO_4_, pH 6.5) and equilibration buffer/cleaning buffer (500 mM Na_2_HPO_4_, pH 6.5). The various antibodies eluted at different positions in these gradients.

### Papain digestion

Either immobilized (Pierce) or soluble (Roche) papain was mixed with purified antibody (5 mg/mL) at a 0.5–2% weight ratio in cutting buffer (20 mM Na_2_HPO_4_, 10 mM EDTA, 20 mM cysteine, pH 7.0) and incubated at 37°C. To follow proteolysis kinetics for each antibody, 50 µg of the digestion reaction was stopped at the desired time (0.5–6 hr) either by addition of 25 mM iodoacetamide (for soluble papain) or removing immobilized papain by filtering. For typical preparative-scale (10–100 mg) Fab production, purified antibody (5 mg/mL) was incubated in cutting buffer with papain (10 µg/mg of antibody) at 37°C for 1–3 hr. It was necessary, however, to pre-determine cutting conditions for each antibody by analytical-scale test reactions. The digestion mixture of Fab and Fc is directly used for complex formation after dialyzing excess iodoacetamide and cysteine out.

### Complex formation and size-exclusion chromatography

For screening of complex formation, purified AdiC or CLC-ec2 (0.2–0.5 mg) was incubated for 1 hr at room temperature with purified full length antibody, purified Fab, or Fab/Fc mixture (3-fold excess for antibody and Fab/Fc mixture or 1.5-fold for purified Fab by OD_280_) in the presence of detergent. The mixture was loaded on a 24-mL bed volume Superdex 200 size-exclusion column (GE healthcare) equilibrated in 100 mM NaCl, 10–20 mM tris-HCl, pH 7.5 with 5 mM DM or 2 mM DDM. Fab-complexes eluted at 11–12 mL elution fraction, with free Fab and Fc eluting around 15–16 mL. Eluted complexes were concentrated to 10 mg/mL and used for crystallization.

## References

[1] Deisenhofer J, Epp O, Miki K, Huber R, Michel H (1985). structure of the protein subunits in the photosynthetic reaction centre of Rhodopseudomonas viridis at 3 A resolution.. Nature.

[2] Ostermeier C, Iwata S, Ludwig B, Michel H (1995). Fv fragment-mediated crystallization of the membrane protein bacterial cytochrome c oxidase.. Nat Struct Biol.

[3] Zhou Y, Morais-Cabral JH, Kaufman A, MacKinnon R (2001). Chemistry of ion coordination and hydration revealed by a K+ channel-Fab complex at 2.0 A resolution.. Nature.

[4] Rasmussen SG, Choi HJ, Rosenbaum DM, Kobilka TS, Thian FS (2007). Crystal structure of the human beta2 adrenergic G-protein-coupled receptor.. Nature.

[5] Fang Y, Jayaram H, Shane T, Kolmakova-Partensky L, Wu F (2009). Structure of a prokaryotic virtual proton pump at 3.2 A resolution.. Nature.

[6] Shaffer PL, Goehring A, Shankaranarayanan A, Gouaux E (2009). Structure and mechanism of a Na+-independent amino acid transporter.. Science.

[7] Dutzler R, Campbell EB, MacKinnon R (2003). Gating the selectivity filter in ClC chloride channels.. Science.

[8] Hibbs RE, Gouaux E (2011). Principles of activation and permeation in an anion-selective Cys-loop receptor.. Nature.

[9] Hunte C, Michel H (2002). Crystallisation of membrane proteins mediated by antibody fragments.. Curr Opin Struct Biol.

[10] Day PW, Rasmussen SG, Parnot C, Fung JJ, Masood A (2007). A monoclonal antibody for G protein-coupled receptor crystallography.. Nat Methods.

[11] Zhou YH, Chen Z, Purcell RH, Emerson SU (2007). Positive reactions on Western blots do not necessarily indicate the epitopes on antigens are continuous.. Immunol Cell Biol.

[12] Sennhauser G, Grutter MG (2008). Chaperone-assisted crystallography with DARPins.. Structure.

[13] Fellouse FA, Esaki K, Birtalan S, Raptis D, Cancasci VJ (2007). High-throughput generation of synthetic antibodies from highly functional minimalist phage-displayed libraries.. J Mol Biol.

[14] Wassef NM, Alving CR, Richards RL (1994). Liposomes as carriers for vaccines.. Immunomethods.

[15] Templeton NS, Lasic DD, Frederik PM, Strey HH, Roberts DD (1997). Improved DNA: liposome complexes for increased systemic delivery and gene expression.. Nat Biotechnol.

[16] Dow SW, Elmslie RE, Willson AP, Roche L, Gorman C (1998). In vivo tumor transfection with superantigen plus cytokine genes induces tumor regression and prolongs survival in dogs with malignant melanoma.. J Clin Invest.

[17] Harlow E, Lane D (1988). Antibodies: A Laboratory Manual.

